# Supplementary figures and images for: Lower Firmicutes abundance in gut microbiota associated with amyloid-β positivity in older adults in Japan as assessed by positron emission tomography
Source: PLoS One. 2025 Oct 29;20(10):e0332801. doi: 10.1371/journal.pone.0332801 (PMC12571266; doi:10.1371/journal.pone.0332801)

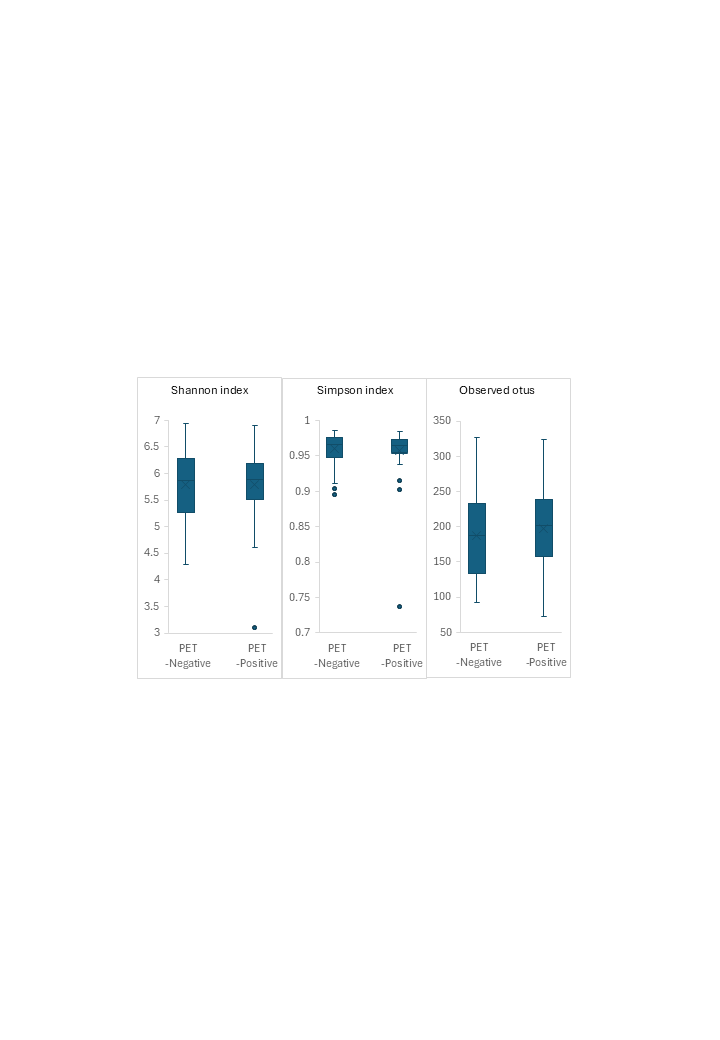

Supplement: S1 Fig — (TIF) [file pone.0332801.s001.tif]

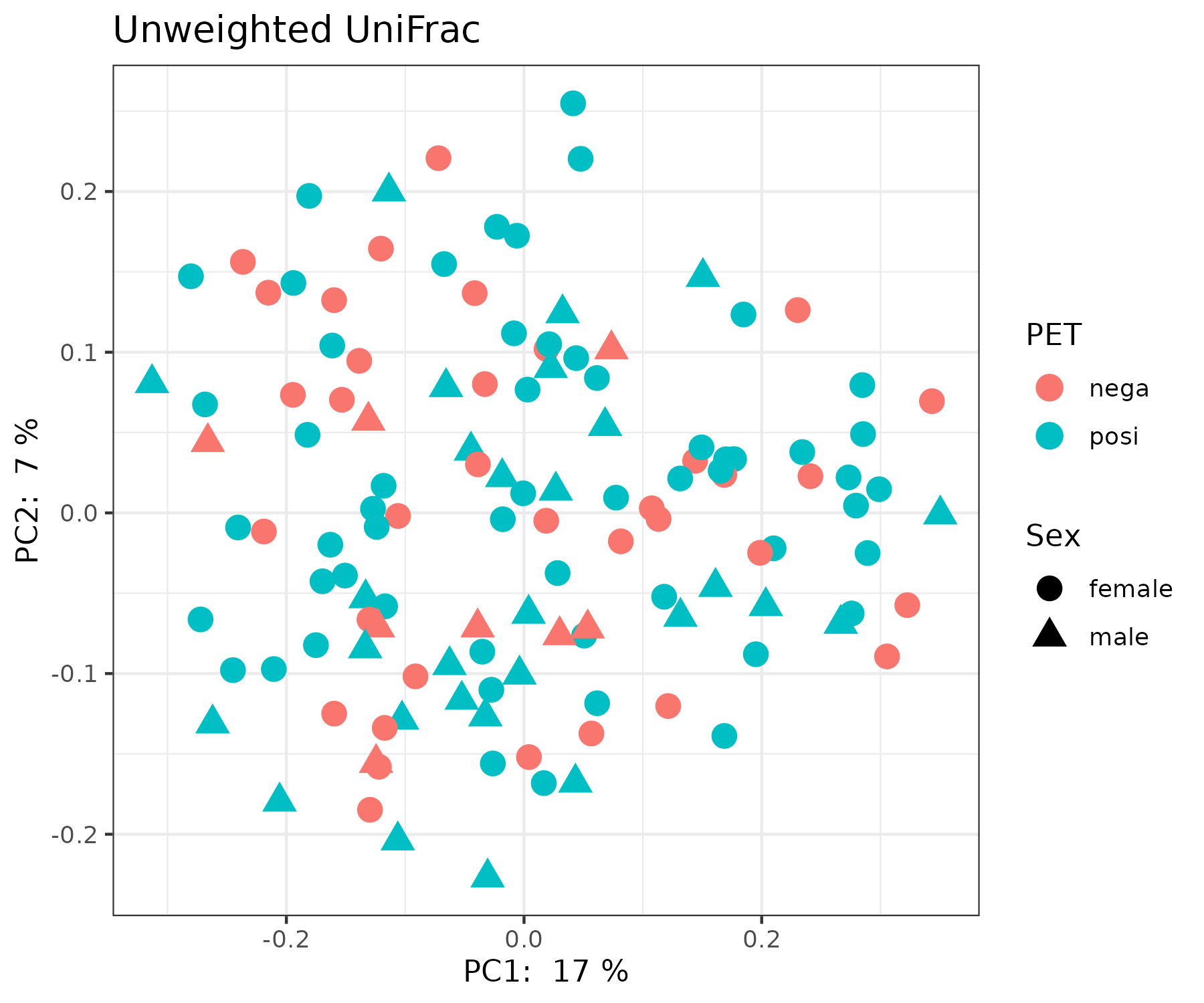

Supplement: S2 Fig — (TIF) [file pone.0332801.s002.tif]
