# Supplementary material for: Lower Firmicutes abundance in gut microbiota associated with amyloid-β positivity in older adults in Japan as assessed by positron emission tomography
Source: PLoS One. 2025 Oct 29;20(10):e0332801. doi: 10.1371/journal.pone.0332801 (PMC12571266; doi:10.1371/journal.pone.0332801)
Supplement: Attachment — Submitted filename: Response to Reviewers.docx [file pone.0332801.s004.docx]

**Reviewer comment (R1-1):**
*“Was 20 minute scan of PET static?”*

**Response:**
Yes, the PET scan used in this study was performed as a static scan. To clarify this point, we have revised the Methods section and now explicitly state that a static PET scan was conducted. The revised sentence reads as follows.

[Changes highlighted in red in the revised manuscript. Line 146]

*"A static PET scan was performed on a PET/computed tomography system (Discovery 710 or Discovery MI; GE Healthcare) for 20 min, starting 90 min after intravenous administration of 185 MBq of Vizamyl to the participants."*

**Reviewer comment (R1-2):**
*“The PET read was only visual and binary (with strict traing and guideline). The Consotium to Establish a Registry for AD is with 88% sensitivity and 88% specificity. This can be potential soruce of misdiagnosis.”*

**Response:**
We thank the reviewer for this insightful comment. In addition to the visual assessment, we have confirmed that quantitative data based on SUVR (Standard Uptake Value Ratio) was available in our dataset. To evaluate the agreement between the visual read and SUVR-based classification (cut-off = 0.58), we conducted an analysis using Cohen’s kappa coefficient. The result showed κ = 0.792, indicating substantial agreement between the two methods. This finding supports the validity of using the visual read in the current analysis. We have now included this analysis and the kappa value in the revised manuscript (Methods and Results sections).

[Changes highlighted in red in the revised manuscript. Line 158]

*“In addition to the visual read, standardized uptake value ratio (SUVR) data were also obtained for each participant. For validation purposes, we conducted a concordance analysis between the binary classification based on the visual read and the SUVR-based classification using a conventional threshold of 0.58.”*

[Changes highlighted in red in the revised manuscript. Line 223]

*“To validate the reliability of the binary classification based on the visual read of amyloid PET images, we compared it with the SUVR-based classification using a cut-off value of 0.58. The concordance between the two methods was substantial, with a Cohen’s kappa coefficient of 0.792.”*

**Reviewer comment (R1-3):**

*“The alpha and beta diversity with either t-test of PCoA test is not well described. More details are needed.”*

**Response:**
Thank you very much for this important comment. In response, we have revised both the Materials and Methods and Results sections to provide more comprehensive descriptions of our diversity analyses.

In the Materials and Methods, we now explicitly specify:

That we evaluated three indices for α diversity: Shannon Index, Simpson Index, and Observed OTUs.

That each index was analyzed using a two-tailed independent t-test.

That Levene’s test was used to assess the assumption of equal variances prior to each t-test, and that all results supported the use of the equal variance assumption.

That for β diversity, we employed unweighted UniFrac distance metrics and visualized the results using PCoA.

That PERMANOVA was performed with 999 permutations using QIIME2 (version 2020.2), and PCoA visualizations were created with R (version 4.2.1) and the ggplot2 package (version 3.5.1).

In the Results, we have now included:

Mean values and p-values for each α diversity index, along with Levene’s test statistics.

Clear reporting of the statistical outcome of the PERMANOVA test.

A summary statement that no significant differences were observed in either α or β diversity between amyloid-positive and amyloid-negative participants.

We believe these additions greatly improve the transparency and reproducibility of our analytical approach.

[Changes highlighted in red in the revised manuscript. Line 34]

*“In addition, α diversity (Shannon, Simpson, Observed OTUs) and β diversity (PCoA based on unweighted UniFrac distances, PERMANOVA) were compared between PET-positive and PET-negative groups.”*

[Changes highlighted in red in the revised manuscript. Line 42]

*“No significant differences were observed in α or β diversity indices between groups.”*

[Changes highlighted in red in the revised manuscript. Line 195]

*“In addition to the primary analyses, we compared α and β diversity between PET-positive and PET-negative participants to explore potential differences in gut microbiota composition.*

*For α diversity, we assessed the Shannon Index, Simpson Index, and Observed OTUs. Each index was compared using a two-tailed independent t-test. Prior to the t-tests, Levene’s test for equality of variances was conducted to confirm the assumption of equal variances.*

*For β diversity, we conducted a Principal Coordinates Analysis (PCoA) using unweighted UniFrac distance matrices. Statistical differences in community structure between the groups were assessed using PERMANOVA with 999 permutations. β diversity analyses were performed using QIIME2 (version 2020.2), and visualizations were generated in R (version 4.2.1) using the ggplot2 package (version 3.5.1).*

*Results were considered statistically significant at P < 0.05. IBM SPSS Statistics version 27 (Armonk, NY, USA) was used for α diversity statistical testing.”*

[Changes highlighted in red in the revised manuscript. Line 286]

*“For α diversity, no significant differences were observed between PET-positive and PET-negative groups across all tested indices. The Shannon Index was 5.79 ± 0.66 for the PET-positive group and 5.78 ± 0.64 for the PET-negative group (t = -0.107, df = 125, p = 0.915; Levene’s test: F = 1.944, p = 0.166). The Simpson Index showed similar results (means not significantly different; 0.957 ± 0.037 v.s. 0.960 ± 0.020, p = 0.557; Levene’s test: F = 0.441, p = 0.508), as did the Observed OTUs (197 ± 56.4 v.s.188 ± 62.4, p = 0.391; Levene’s test: F = 1.174, p = 0.281) (S1 Fig).*

*For β diversity, PCoA based on unweighted UniFrac distances revealed no distinct clustering between PET-positive and PET-negative groups. PERMANOVA analysis further confirmed that there were no significant differences in overall microbial community structure between the two groups (PERMANOVA p = 0.661) (S2 Fig).”*

**Reviewer comment (R1-4):**

*“CDR>=0.5 is indicative of some cognition impairment. The percentage amyloid positivity 38.3% vs 20.2% could be biologically meaningful and statistially significant. This should be examined. The same is true for each item listed in Table 1”*

**Response:**

Thank you very much for your insightful suggestion. Although Table 1 was originally presented for descriptive purposes only, we agree that a statistical comparison between amyloid PET-positive and PET-negative groups can provide additional interpretative value.

Therefore, we conducted appropriate statistical tests (independent t-tests for continuous variables and chi-square tests for categorical variables) to examine differences between the two groups across the characteristics listed in Table 1.

The results have now been added to Table 1 (including p-values), and a brief summary has been included in the Results section. These analyses are presented for exploratory purposes only and do not alter the primary outcomes of the study.

[Changes highlighted in red in the revised manuscript. Line 171]

*“As a preliminary step, group comparisons of participant characteristics between amyloid PET-positive and PET-negative individuals were performed using independent-samples t-tests for continuous variables and chi-square tests (or Fisher’s exact tests where appropriate) for categorical variables.”*

[Changes highlighted in red in the revised manuscript. Line 212]

*“The final sample included 40 men (29.4%) and 96 women. The participants ranged in age from 68 to 86 years, with a mean of 79.2 years (SD = 4.0). The mean MMSE score was 28.3 (SD = 1.9), indicating generally preserved global cognitive function. However, 26.5% of participants had a CDR score of ≥ 0.5, suggestive of mild cognitive impairment.*

*Amyloid PET was positive in 47 participants (34.6%) and negative in 89 (65.4%). Comparisons between PET-positive and PET-negative groups revealed no significant differences in sex, age, or history of hypertension or diabetes mellitus. However, the PET-positive group had significantly fewer years of education (mean = 12.2 vs. 13.4 years, p = 0.006), lower MMSE scores (mean = 27.7 vs. 28.6, p = 0.012), and a higher proportion of individuals with CDR ≥ 0.5 (38.3% vs. 20.2%, p = 0.023) (Table 1).”*

**Reviewer comment (R1-5):**

*“there are 6 phyla, and the binary logistic regression result significance may need to correct for mulitple comparison (6 of them). If not, justification is needed (firmicutes is reported in some other studies in other populations?)”*

**Response:**

We appreciate the reviewer’s thoughtful comment regarding the potential need for multiple comparison correction in the phylum-level analyses. In our study, the primary hypothesis concerned the association between the Firmicutes phylum and amyloid PET positivity, as suggested by previous literature [3]. Therefore, Firmicutes was treated as the primary phylum of interest, and the other five phyla were included as exploratory variables to provide a comprehensive overview. In line with this hypothesis-driven approach, we did not apply multiple comparison correction to the phylum-level analyses. We have now clarified this rationale in the Statistical analysis section of the Methods. We believe this approach appropriately balances hypothesis testing and exploratory analysis, while making the rationale for the statistical treatment transparent to the reader.

[Changes highlighted in red in the revised manuscript. Line 185]

*“We did not apply multiple comparison correction to the six phyla tested because our primary hypothesis focused on the phylum Firmicutes, which has been associated with amyloid pathology in previous literature [3]. Other phyla were included as exploratory variables to provide a comprehensive picture, but the results should be interpreted with caution.”*

**Reviewer comment (R2-1-1):**

*“Lack of genus-level analysis despite using 16S Amplicon gene sequencing.”*

**Response:**

We thank the reviewer for the suggestion to explore the association between amyloid PET positivity and bacterial taxa at the genus level. In response, we conducted additional genus-level analyses. For each genus, we categorized participants into higher or lower than the median relative abundance groups, performed binomial logistic regression adjusted for age, sex, and recent antibiotic use, and applied false discovery rate correction (Benjamini–Hochberg procedure) to account for multiple comparisons.

The results of these analyses have been added as Table 3 in the Results section. After FDR adjustment, no genera showed statistically significant associations with amyloid PET positivity. The Methods (Statistical analysis section), Results, and Discussion have been updated to describe the analytical approach, present the findings, and discuss the implications of the absence of significant genus-level associations.

We believe these additions provide a more comprehensive understanding of the gut microbiota–amyloid PET relationship and transparently convey both significant and non-significant findings.

[Changes highlighted in red in the revised manuscript. Line 30]

*“Taxonomic composition was evaluated at both the phylum and genus levels; participants were classified into above- and below-median groups based on the relative abundance of each taxon.”*

[Changes highlighted in red in the revised manuscript. Line 33]

*“For genus-level analyses, p-values were further corrected for multiple comparisons. In addition, α diversity (Shannon, Simpson, Observed OTUs) and β diversity (PCoA based on unweighted UniFrac distances, PERMANOVA) were compared between PET-positive and PET-negative groups.”*

[Changes highlighted in red in the revised manuscript. Line 41]

*“At the genus level, no taxon remained significant after correction for multiple comparisons. No significant differences were observed in α or β diversity indices between groups.”*

[Changes highlighted in red in the revised manuscript. Line 174]

*“For every bacterial phylum and genus, a χ-square test was performed for the association between the binary variable for the bacterial relative abundance (i.e., bacterial relative abundance is less or greater than the median among participants) and the PET result (positive/negative). Subsequently, for each phylum and genus, a binomial logistic regression analysis adjusted for age [16], sex, and use of antibiotics within 2 weeks was performed, with higher or lower than the median relative abundance of each phylum or genus as the independent variable and the amyloid PET result as the dependent variable, i.e we used binary variables instead of continuous proportion data as explanatory variables in logistic regression.”*

[Changes highlighted in red in the revised manuscript. Line 183]

*“For genus-level analyses, p-values were additionally adjusted for multiple comparisons using the false discovery rate (FDR) method (Benjamini–Hochberg procedure). We did not apply multiple comparison correction to the six phyla tested because our primary hypothesis focused on the phylum Firmicutes, which has been associated with amyloid pathology in previous literature [3]. Other phyla were included as exploratory variables to provide a comprehensive picture, but the results should be interpreted with caution.”*

[Changes highlighted in red in the revised manuscript. Line 265]

*“At the genus level, odds ratios for amyloid PET positivity were calculated for each genus using the same dichotomization approach (higher vs. lower than the median relative abundance) and adjusting for age, sex, and antibiotics use (Table 3). Several genera, including Clostridium, Ruthenibacterium, and Dorea, showed nominally significant associations (p < 0.05). However, none of the associations remained statistically significant after controlling for multiple comparisons using the false discovery rate (FDR) correction.”*

[Changes highlighted in red in the revised manuscript. Line 272]

*“Table 3. Odds ratios of amyloid PET positivity according to higher (reference group) or lower than the median relative abundance of intestinal bacteria at the genus level”*

[Changes highlighted in red in the revised manuscript. Line 275]

*“Adjusted for sex, age, antibiotics use. β: Regression coefficient, OR: Odds ratio, CI: Confidence interval. The reference group for each genus is defined as participants with a relative abundance above the median value. Odds ratios (ORs) indicate the likelihood of amyloid PET positivity in participants with a relative abundance below the median compared to those in the reference group. † These are taxonomic labels from genome-based databases representing uncultured or unclassified genera. Genera with suffixes such as "_A", "_B", or "_Q" represent phylogenetically distinct clades within broader taxonomic groups, based on genome-based databases classifications. Genera detected in less than 50% of participants (i.e., those with a median relative abundance of zero) were excluded from the analysis.”*

[Changes highlighted in red in the revised manuscript. Line 302]

*“At the genus level, several genera within Firmicutes and other phyla showed nominal associations with PET positivity; however, none remained statistically significant after FDR correction. These findings suggest that the observed phylum-level association may not be driven by a single genus with a strong effect, but rather by broader compositional features within the Firmicutes phylum. The consistency of our phylum-level results with previous studies in both Japanese and American populations [3]—despite differences in diet and gut microbiota composition—underscores the potential relevance of Firmicutes to amyloid-β accumulation.”*

**Reviewer comment (R2-1-2):**

*“Alpha (Shannon but also Simpson, Observed) and beta diversity plots should also be presented irrespective if they are negative or positive results.”*

**Response:**

We appreciate the Reviewer’s suggestion. In response, we have now added figures to illustrate both α and β diversity results. Specifically, Supplementary Figure 1 presents boxplots of the Shannon, Simpson, and Observed OTUs indices, showing no significant differences between PET-positive and PET-negative groups. In addition, Supplementary Figure 2 shows a principal coordinate analysis (PCoA) plot of β diversity based on unweighted UniFrac distances, also indicating no clear separation between the two groups. These supplementary figures complement the statistical results described in the Results section and provide a visual representation of the diversity analyses, irrespective of the lack of significant differences.

[Changes highlighted in red in the revised manuscript. Line 488]

*“S1 Fig. Comparison of alpha diversity indices (Shannon, Simpson, Observed OTUs) between PET-positive and PET-negative groups.”*

[Changes highlighted in red in the revised manuscript. Line 490]

*“S2 Fig. Principal coordinate analysis (PCoA) plot of β diversity based on unweighted UniFrac distances between amyloid PET-positive and -negative participants.”*

**Reviewer comment (R2-2):**

*“2. Missing public data availability — violates PLOS ONE policy*

*The manuscript does not provide access to any raw or processed microbiome data, such as:*

*FASTQ files from the sequencing run*

*Feature/ASV tables or taxonomic annotations*

*This is a clear violation of PLOS ONE's data availability policy:*

*“Authors must follow standards and practice for data deposition in publicly available resources including those created for gene sequences, microarray expression, structural studies, and similar kinds of data.”*

*The authors must deposit their data in a public repository such as NCBI, ENA, or any other and provide accession numbers in the revised manuscript. Please check journal guidelines for clinical data.”*

**Response:**

Following your valuable advice, we have completed the data registration.

The data is registered with DDBJ, managed by the National Institute of Genetics, and the Bioproject ID is PRJDB37280. The data is set to private until the paper is accepted. However, it will be made public after 2027/8/27, even if the paper has not been accepted by that date.

[Changes highlighted in red in the revised manuscript. Line 411]

*“Data Availability*

*The sequencing data reported in this paper are available from DDBJ Sequence Read Archive (DRA) (BioProject PRJDB37280). These datasets are publicly available.*

*Other clinical datasets analyzed in this study (including amyloid PET results, demographic variables, and health-related parameters) contain potentially sensitive personal information. According to the policy of the Research Ethics Committee of the Tokyo Metropolitan Institute for Geriatrics and Gerontology, these data cannot be shared publicly. Data access requests can be directed to the Administrative Office of the Research Ethics Committee (E-mail: kenkyurinri@tmghig.jp), and will be considered for researchers who meet the criteria for access to confidential data.”*

**Editorial comment-1 (E-2)**

*“Please provide an amended Competing Interests Statement that explicitly states this commercial funder, along with any other relevant declarations relating to employment, consultancy, patents, products in development, marketed products, etc.*

*Within this Competing Interests Statement, please confirm that this does not alter your adherence to all PLOS ONE policies on sharing data and materials by including the following statement: "This does not alter our adherence to PLOS ONE policies on sharing data and materials.” (as detailed online in our guide for authors http://journals.plos.org/plosone/s/competing-interests). If there are restrictions on sharing of data and/or materials, please state these. Please note that we cannot proceed with consideration of your article until this information has been declared.*

*Please include your amended Competing Interests Statement within your cover letter. We will change the online submission form on your behalf.”*

**Response:**

Competing Interests Statement

This study was funded by Otsuka Pharmaceutical Co., Ltd., Shiga, Japan. SS and HK received research funding from Otsuka Pharmaceutical Co., Ltd. NoK and YK are employees of Otsuka Pharmaceutical Co., Ltd. and contributed mainly to the microbiome analyses and provided advice on the manuscript. The initial study design was discussed with representatives from Otsuka Pharmaceutical Co., Ltd. during the project planning stage. The funder had no role in the data collection, statistical analysis, or the decision to publish. The authors declare no additional competing interests. This does not alter our adherence to PLOS ONE policies on sharing data and materials.

**Editorial comment-2 (E-3)**

*“3. We note that you have indicated that there are restrictions to data sharing for this study. PLOS only allows data to be available upon request if there are legal or ethical restrictions on sharing data publicly. For more information on unacceptable data access restrictions, please see http://journals.plos.org/plosone/s/data-availability#loc-unacceptable-data-access-restrictions.*

*Before we proceed with your manuscript, please address the following prompts:*

*a) If there are ethical or legal restrictions on sharing a de-identified data set, please explain them in detail (e.g., data contain potentially identifying or sensitive patient information, data are owned by a third-party organization, etc.) and who has imposed them (e.g., a Research Ethics Committee or Institutional Review Board, etc.). Please also provide contact information for a data access committee, ethics committee, or other institutional body to which data requests may be sent.*

*b) If there are no restrictions, please upload the minimal anonymized data set necessary to replicate your study findings to a stable, public repository and provide us with the relevant URLs, DOIs, or accession numbers. For a list of recommended repositories, please see*

*https://journals.plos.org/plosone/s/recommended-repositories. You also have the option of uploading the data as Supporting Information files, but we would recommend depositing data directly to a data repository if possible.*

*We will update your Data Availability statement on your behalf to reflect the information you provide.”*

**Response:**

We appreciate the editor’s comments regarding data availability. In response, we have revised the Data Availability statement to clarify the accessibility of our datasets.

Specifically, the sequencing data reported in this paper are available from DDBJ Sequence Read Archive (DRA) (BioProject PRJDB37280).

For the clinical datasets (amyloid PET results, demographic and health-related parameters), public sharing is not permitted due to ethical restrictions. According to the policy of the Research Ethics Committee of the Tokyo Metropolitan Institute for Geriatrics and Gerontology, even anonymized datasets cannot be made openly available because the research team retains the key linking participant IDs to personal identifiers. To address this, we have indicated that requests for access will be considered by the Research Ethics Committee. Contact information for the committee has been added.

[Changes highlighted in red in the revised manuscript. Line 411]

*“Data Availability*

*The sequencing data reported in this paper are available from DDBJ Sequence Read Archive (DRA) (BioProject PRJDB37280). These datasets are publicly available.*

*Other clinical datasets analyzed in this study (including amyloid PET results, demographic variables, and health-related parameters) contain potentially sensitive personal information. According to the policy of the Research Ethics Committee of the Tokyo Metropolitan Institute for Geriatrics and Gerontology, these data cannot be shared publicly. Data access requests can be directed to the Administrative Office of the Research Ethics Committee (E-mail: kenkyurinri@tmghig.jp), and will be considered for researchers who meet the criteria for access to confidential data.”*

Editorial comment-3 (E-4)

*“4. Please include captions for your Supporting Information files at the end of your manuscript, and update any in-text citations to match accordingly. Please see our Supporting Information guidelines for more information: http://journals.plos.org/plosone/s/supporting-information.”*

**Response:**

We have revised the manuscript to comply with the PLOS ONE Supporting Information guidelines. Specifically, all in-text citations now refer to “S1–S4 Files”, “S1 Fig”, “S2 Fig”. Captions for the Supporting Information files or figures have been placed at the end of the manuscript, following the References section, as requested.

[Changes highlighted in red in the revised manuscript. Line 487]

*“Supporting Information*

*S1 Fig. Comparison of alpha diversity indices (Shannon, Simpson, Observed OTUs) between PET-positive and PET-negative groups.*

*S2 Fig. Principal coordinate analysis (PCoA) plot of β diversity based on unweighted UniFrac distances between amyloid PET-positive and -negative participants.*

*S1 File. Ethics approval certificate (Japanese, issued January 27, 2020).*

*S2 File. English translation of ethics approval certificate (issued January 27, 2020).*

*S3 File. Ethics approval certificate (Japanese, issued October 7, 2021).*

*S4 File. Ethics approval certificate with English translation and signature (issued October 7, 2021).”*
